# Retroperitoneal and pelvic schwannoma/neurofibroma resection: surgical strategies and outcomes in a neurosurgical cohort

**DOI:** 10.1007/s00701-025-06745-8

**Published:** 2025-12-08

**Authors:** Bilal Younes, Dorothee Mielke, Veit Rohde, Tammam Abboud

**Affiliations:** 1 Department of Neurosurgery, University Medical Center Göttingen, Robert-Koch-Straße 40, 37075 Göttingen, Germany; 2 Department of Neurosurgery, University Hospital Augsburg, Augsburg, Germany

**Keywords:** Surgical strategies, Schwannoma, Retroperitoneal and pelvic schwannoma

## Abstract

**Background:**

Retroperitoneal and pelvic schwannomas and neurofibromas account about 10% of all retroperitoneal tumors. These tumors are almost invariably benign and slow growing. They are either asymptomatic or cause radicular or abdominal pain. The radiologic findings cannot distinguish schwannomas from other retroperitoneal neoplasms. To our knowledge, this study is the first neurosurgical series of this size to employ intraoperative electrophysiological monitoring during resection of schwannomas and neurofibromas arising in the retroperitoneal and pelvic regions.

**Methods:**

A retrospective study conducted at the University Hospital Göttingen from 2015 to 2024 included 13 patients who underwent surgical treatment for schwannomas and neurofibromas arising in the retroperitoneal and pelvic regions. The study incorporated detailed surgical descriptions of the resection techniques and the approaches used for these tumors.

**Results:**

The mean age was 51 ± 12 years. Symptomatic presentations included abdominal discomfort in 6 patients (46%), unilateral radicular pain in 5 patients (38%), and 4 patients (31%) were asymptomatic. Tumors exhibited a mean diameter of 6.2 ± 2.9 cm (range: 3.3–14 cm). Anatomic distribution included 7 cases (54%) in the presacral region, 5 cases (38%) in the lesser pelvis, and 4 cases (31%) involving the L5 or S1 neuroforamen with extension into the ventral prevertebral space. Transretroperitoneal approaches were utilized in 8 cases (62%), while 5 (38%) underwent transperitoneal resection. Gross total resection was achieved in 10 patients (77%). In one patient, a transient intraoperative decline in sphincter MEPs was observed, with a 48% drop in amplitude, followed by full postoperative recovery. Direct electrical stimulation of the tumor capsule elicited active motor responses in 5 patients (38%). In 3 of these cases, complete resection was not feasible due to intraoperative changes in MEPs signals. The mean operative duration was 271.8 ± 64.5 min (range: 180–400 min), with a mean blood loss of 700 ± 400 mL. Postoperatively, no motor or sensory deficits occurred, and symptoms resolved within one week. The mean hospital stay was 9.2 ± 3.5 days (range: 5–15 days). Histopathology confirmed benign tumors in all cases: 8 schwannomas (62%), 3 neurofibromas (23%), and 1 ganglioneuroma (8%). No recurrences were observed during a mean follow-up period of 24 ± 6 months.

**Conclusion:**

Surgical resection of retroperitoneal and pelvic schwannomas and neurofibromas, while technically challenging, is safe and effective when performed by experienced surgeons and multidisciplinary preoperative planning. None of our patients experienced postoperative complications, which may, in part, be attributable to the use of intraoperative neuromonitoring. However, comparative and prospective studies are recommended to further validate these findings.

## Introduction

Benign tumors of the peripheral nerve sheath are broadly classified as schwannomas and neurofibromas [2]. These tumors may develop along any cranial, peripheral, or autonomic nerve, but they most frequently involve the head, neck, and extremities. Retroperitoneal and pelvic schwannomas account for only about 10% of all retroperitoneal tumors and roughly 1–3% of schwannomas overall [4, 11]. Schwannomas typically present in the third to fifth decades of life and are usually solitary [14]. They occur most often sporadically. However, familial tumor syndromes can be involved. For example, alterations in the Neurofibromatosis type 2 (NF2) gene predispose to schwannomas, whereas Neurofibromatosis type 1 (NF1) mutations are linked to neurofibromas [7, 15]. These tumors are almost invariably benign and well-circumscribed; malignant transformation is exceedingly rare [9]. Because schwannomas are slow growing, they may reach considerable size before detection. Patients with pelvic or retroperitoneal schwannomas often have minimal or nonspecific symptoms until the tumor grows large [8]. Symptoms, when present, typically relate to mass effect, for example, vague or abdomen discomfort, gastrointestinal or urinary compression, or even sciatica, depending on the tumor’s location [16]. Many cases are discovered incidentally on imaging performed for unrelated reasons. Computed tomography (CT) and/or magnetic resonance imaging (MRI) generally shows a well-defined soft-tissue mass, sometimes with cystic degeneration or heterogeneous enhancement [9]. However, these radiologic features are not unique to schwannoma, and preoperative imaging alone often cannot distinguish a schwannoma from other retroperitoneal neoplasms. Definitive diagnosis thus relies on tissue evaluation. In practice, CT guided needle biopsy is frequently performed for retroperitoneal masses, as this approach is safe and effective for obtaining a histologic diagnosis [12]. Establishing that a lesion is a benign schwannoma preoperatively is important, because it allows the surgical team to plan a more conservative, nerve-sparing resection, in contrast to the wide en-bloc excision typically indicated for a high-grade sarcoma [6, 17]. The choice between resection and conservative control of the tumor must be individualized, taking into account multiple factors such as patient age, symptomatology, tumor size, anatomical location, and most importantly, pathological confirmation [3, 13]. Complete surgical excision remains the gold standard treatment for schwannomas [5, 10]. Given their encapsulated, benign nature, complete resection is typically curative and local recurrence is uncommon [2]. However, pelvic and retroperitoneal schwannomas often arise adjacent to major vessels (aorta, vena cava, iliac vessels) and neural plexuses (e.g. sacral or hypogastric plexus), which can complicate surgery [1, 11, 14, 16]. These operations frequently require a multidisciplinary approach (for example, involving neurosurgeons, vascular surgeons, visceral surgeons or gynecologic) to achieve safe resection [5, 16]. Typical schwannomas display a biphasic histologic pattern. Antoni A areas consist of compact, spindle-shaped Schwann cells arranged in fascicles, while Antoni B areas are less cellular and myxoid. A “cellular” variant of schwannoma, seen in most large retroperitoneal and pelvic schwannomas, is composed predominantly of densely packed spindle cells without distinct Antoni A or B regions. Immunohistochemically, schwannoma cells are characteristically strongly and diffusely positive for S100 protein, reflecting their neural crest derivation [7, 17].

Most of the available literature consists of isolated case reports or small series authored by general surgeons, none of which have employed intraoperative electrophysiological monitoring [2, 4, 11–14, 16]. To our knowledge, this study is the first neurosurgical series of this size to employ intraoperative electrophysiological monitoring during resection of schwannomas and neurofibromas arising in the retroperitoneal and pelvic regions and to systematically describe the procedural workflow and tumor characteristics. We analyze clinical presentations, imaging and pathological findings (including immunohistochemical profiles), and treatment modalities with emphasis on surgical approach. Our goal is to evaluate and characterize the safety, feasibility, and surgical outcomes of resecting schwannomas and neurofibromas arising in the retroperitoneal and pelvic regions.

## Methods

### Patients

This retrospective study included patients treated for schwannomas and neurofibromas located in the retroperitoneal and pelvic regions at the University Hospital Göttingen between 2015 and 2024. Only patients who underwent surgical resection with available postoperative follow-up were included. Patients were excluded if they were managed conservatively, had incomplete follow-up documentation, or lacked intraoperative electrophysiological monitoring data.

### Preoperative course

All patients underwent standardized preoperative evaluations, including routine hematologic and biochemical laboratory studies. Cross-sectional imaging with contrast-enhanced CT with angiography (CTA) and contrast-enhanced MRI was performed to delineate tumor size, vascular relationships, and anatomical involvement with adjacent organs. Each case was reviewed prospectively in a dedicated tumor board involving specialists from different departments. This collaborative approach ensured consensus on surgical goals, risk stratification, and intraoperative strategies. For the earlier patients in this study, pre-operative biopsy was not standard practice; however, it was implemented for the last seven cases, where a CT-guided core-needle biopsy was performed to confirm the diagnosis. Histopathological analysis determined tumor subtype, WHO grade, and immunohistochemical results, which guided individualized surgical planning.

### Surgical management

In most cases, retroperitoneal and pelvic schwannomas were resected via a midline laparotomy with longitudinal umbilical incision. In select cases, a Pfannenstiel incision was utilized. One patient underwent a lateral decubitus position with a trans-retroperitoneal approach. The surgical approach was determined by tumor size and location: a transperitoneal approach for larger, medially located tumors, and a retroperitoneal approach for smaller, lateral ones.

### Operative technique

The patient was placed in supine position. Prophylactic antibiotics were administered.

#### Ureteral Stenting

Bilateral or ipsilateral double-J ureteral stents were placed preoperatively by urology to facilitate intraoperative identification and mobilization of the ureters, minimizing injury during tumor dissection.

#### Neuromonitoring

For Motor-evoked potentials (MEPs) and electromyography (EMG), needle electrodes were inserted into multiple muscle groups, including the quadriceps, biceps femoris, tibialis anterior, gastrocnemius, abductor hallucis, abductor digiti minimi, gluteus maximus, and internal anal sphincter. A muscle group located above the surgical level was selected as a control.

#### Surgical approaches

In the intraperitoneal approach, a midline laparotomy with longitudinal umbilical incision was performed, followed by entry into the abdominal cavity. Wound edge retraction and placement of a self-retaining abdominal retractor system were completed. The small bowel and cecum were mobilized from the retroperitoneum, exposing the tumor as a retroperitoneal bulge. The small bowel was packed into the upper abdomen and secured with a self-retaining system. For the retroperitoneal approach, the muscular layers of the abdominal wall (external oblique, internal oblique, and transversus abdominis) were sequentially split along the direction of their fibers. The underlying transversalis fascia was incised to enter the retroperitoneal space. The peritoneal sac was then meticulously mobilized and retracted anteromedially, providing direct exposure to the psoas muscle, iliac vessels, and ureter. This dissection created a direct corridor to the retroperitoneal tumor, which was carefully dissected from its surrounding neurovascular structures without entering the peritoneal cavity.

#### Tumor resection

**Fig. 1:**
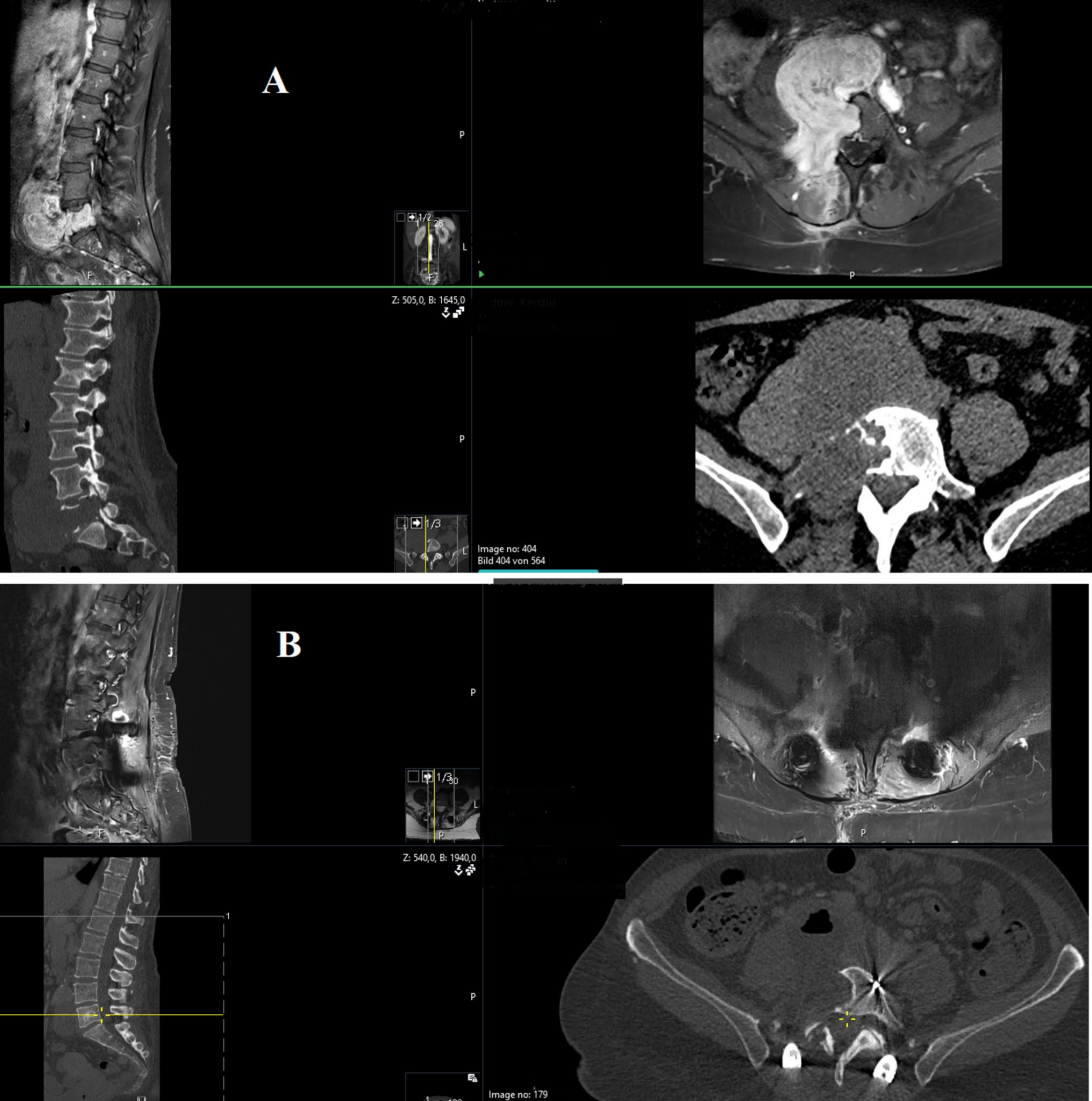
**A** Preoperative contrast-enhanced MRI and CT imaging revealed a large, heterogeneously enhancing tumor with extensive bony destruction involving the facet joint, pedicle, and approximately 50% of the L5 vertebral body. **B **Postoperative contrast-enhanced MRI and CT following right-sided dorsal hemilaminectomy and transpedicular tumor resection to the anterior margin of the L5 vertebral body, with a second-stage transretroperitoneal resection of the remaining tumor. The MRI shows no residual tumor, and the CT confirms correct placement of pedicle screws in L4 and S1. (Carbon Fiber–Reinforced Pedicle Screws)

Critical structures, including the ureters, internal iliac artery, and adjacent vasculature, were meticulously identified and dissected free. Once the tumor was exposed, we focused on identifying both the distal and proximal ends of the lesion, using nerve stimulation to track fascicular pathways. The tumor capsule was stimulated using a neurostimulator to map functional neural tissue. Following capsular coagulation and incision, the tumor should carefully be evacuated from its internal cavity using a combination of bipolar and blunt dissection. This intracapsular decompression allowed for gradual reduction of tumor volume, improving visualization of the tumor-neural interface and minimizing traction on adjacent structures, which reduced the risk of neurological complications. In situations where CT-guided biopsy is not feasible, intraoperative frozen section pathology was performed. After internal evacuation, the residual capsular walls were systematically resected under continuous neuromonitoring guidance. Direct stimulation of the remaining capsule was repeated to confirm the absence of motor responses prior to final resection to avoid postoperative deficits. Tumor extensions into neural foramina were intentionally left unresected to prevent nerve injury (Fig. 1). En bloc resection was avoided due to the high risk of irreversible motor deficits, particularly in young patients with slow-growing tumors. A Robinson drain was placed in the right lower abdomen. Closure proceeded in layers: fascia, subcutaneous tissue, and skin.

#### Intraoperative monitoring

**Fig. 2:**
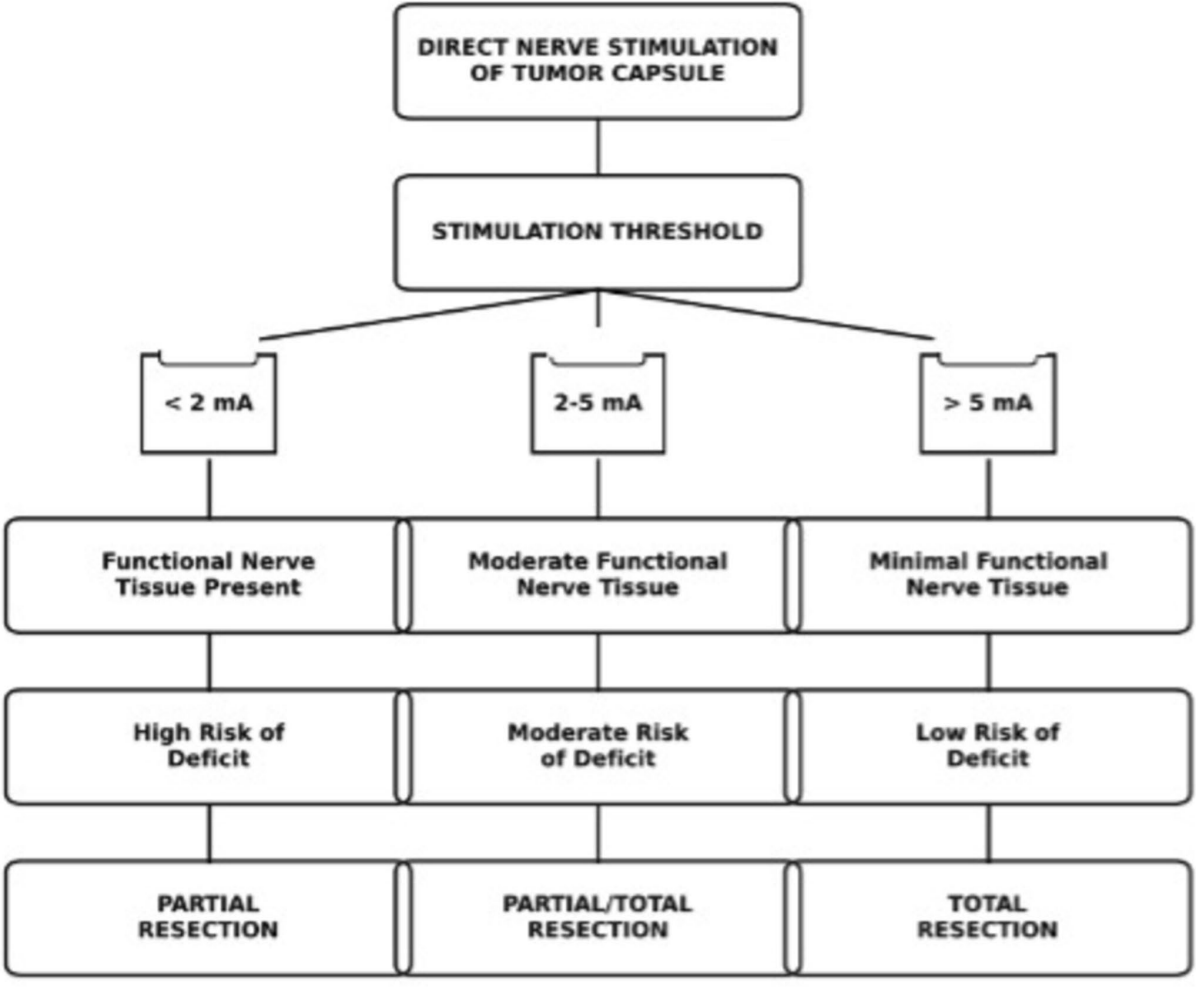
Clinical decision algorithm based on nerve stimulation threshold

The MEPs, and both spontaneous and triggered EMG using electrical stimulation were continuously monitored throughout the resection procedure to guide the extent of neural dissection. These monitoring techniques were employed to assess the functional integrity of motor and sensory pathways during the intervention, helping to minimize the risk of postoperative neurological deficits (Fig. 2).

### Postoperative course

All patients were transferred to the intensive care unit (ICU) for 24 h postoperatively. Compression stockings were applied prophylactically to reduce the risk of lymphedema. Prophylactic therapy with weight-adjusted low-molecular-weight heparin (e.g., enoxaparin) was initiated 12 h after surgery to prevent thromboembolic complications. Laboratory parameters, including hemoglobin levels, were evaluated on postoperative day 1 to assess for bleeding. Routine postoperative diagnostic imaging (e.g., CT or MRI) was deemed unnecessary unless clinically indicated by hemodynamic instability or neurologic deficits. Abdominal sonography (ultrasound) is a reliable initial modality to assess for free intra-abdominal fluid collections in the postoperative period. The surgical drain was removed on postoperative day 2.

### Follow-up assessments

All patients underwent contrast-enhanced MRI of the abdomen 3 months postoperatively to evaluate for residual or recurrent disease. For confirmed WHO grade I schwannomas and neurofibromas, annual contrast-enhanced abdominal MRI surveillance was instituted to monitor long-term disease progression.

### Statistical methods

Descriptive statistics summarized continuous variables (mean, range). Kaplan–Meier analysis assessed recurrence-free survival. Inferential analyses included: Mann–Whitney U test (BMI vs. residual tumor status); Spearman's rank correlation (tumor size vs. blood loss); Fisher's exact test (residual tumor vs. location); and chi-square test with Monte Carlo simulation (symptom type vs. location). Data were managed in a Microsoft Excel database (Microsoft Corp) and exported to SPSS for statistical analysis, which was performed using IBM SPSS Statistics for Windows, Version 27.0 (IBM Corp, Armonk, NY, USA).

### Ethics Statement

Ethical approval was secured (ethical commission of University Hospital Göttingen, application number: 16/8/25), aligning with the 1964 Declaration of Helsinki and its amendments. All procedures adhered to local and institutional laws and data protection regulations.

## Results

The study cohort comprised 13 patients (4 males [30%], 9 females [70%]; gender ratio 1:2.3) with a mean age of 51 ± 12 years (range: 25–62 years). Four patients (31%) were asymptomatic, with tumors discovered incidentally during routine examinations or pregnancy-related imaging. Symptomatic presentations included abdominal discomfort in 6 patients (46%), and unilateral radicular pain in 5 patients (38%). Rare symptoms (each < 7%) included urinary retention, abdominal distension, and motor weakness.Tumors exhibited a mean diameter of 6.2 ± 2.9 cm (range: 4–16 cm), with 7 cases (54%) located in the presacral region, 5 cases (38%) in the lesser pelvis, and 4 cases (31%) in the L5 or S1 neuroforamen extending into the ventral prevertebral space. Notably, 10/13 tumors (77%) measured ≤ 9.4 cm, aligning with the typical size range for these lesions. Preoperative CT-guided needle biopsy was performed in 7 patients (54%), with one case complicated by radicular pain and paresthesia. Surgical approaches included midline laparotomy in 9 patients (69.2%) and Pfannenstiel incision in 3 patients (23.1%). A trans-retroperitoneal approach was utilized in 8 cases (62%), while 5 (38%) underwent trans-intraperitoneal resection. Approaches were performed by visceral surgeons in 12 cases (92%) and gynecologic surgeons in 1 case (8%). A total resection was achieved in 10 patients (77%), with intentional residual tumor left in 3 cases (23%) due to intraoperative motor responses during direct nerve stimulation. No vascular injuries occurred. In one patient, a transient intraoperative decline in sphincter MEPs was observed, with a 48% drop in amplitude, followed by full postoperative recovery. Direct electrical stimulation of the tumor capsule elicited active motor responses in 5 patients (38%). In 3 of these cases, complete resection was not feasible due to intraoperative changes in MEPs signals, necessitating intentional subtotal resection to preserve neurological function.

**Fig. 3:**
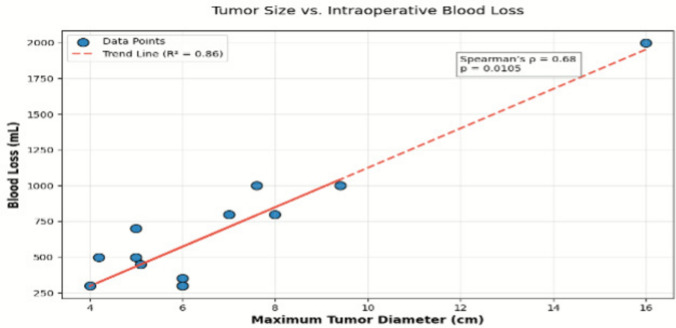
Scatterplot demonstrating a significant positive correlation between maximum tumor diameter (cm) and intraoperative blood loss

The mean operative duration was 271.8 ± 64.5 min (range: 180–400 min), with mean blood loss of 700 ± 400 mL. No statistically significant correlation was observed between tumor size and operative duration (Spearman’s ρ = 0.52, *p* = 0.07). A significant correlation was observed between tumor size and intraoperative blood loss (Spearman’s ρ = 0.68, *p* = 0.0005; Fig. 3). Tumors exceeding 7 cm in diameter were associated with a nearly fourfold increase in blood loss compared to smaller lesions. Postoperatively, no motor or sensory deficits were observed, and the patient's preoperative symptoms and local wound pain resolved within one week. The mean hospital stay was 9.2 ± 3.5 days (range: 5–15 days). The pathology confirmed benign tumors in the entire cohort: 8 schwannomas (62%), 3 neurofibromas (23%), and 1 ganglioneuroma (8%), all classified as WHO Grade I. In one case, histopathology revealed an encapsulated chronic hematoma (non-neoplastic), which was excluded from tumor subtype categorization. Residual tumor (2 × 1.8 cm, 2.6 × 1.4 cm and 5 × 4 cm) was identified in 3 patients on 3-month follow-up MRI, though none showed progression at 1 years. No recurrences were observed during a mean follow-up of 24 ± 6 months, and no adjuvant therapies were required. Detailed demographic, surgical, and histopathologic features of patients undergoing resection of pelvic and retroperitoneal nerve sheath tumors are shown in Table 1. Immunohistochemical findings for schwannomas are summarized in Table 2. In patients with neurofibromas, S100 and SOX10 expression was observed in all cases. The Ki67 proliferation index was consistently low (3%) across all three patients, and PHH3 immunohistochemistry highlighted rare mitotic figures (individual mitoses). CD34 and vimentin were negative in tumor cells. Detailed demographic, surgical, and histopathologic features of patients undergoing resection of pelvic and retroperitoneal nerve sheath tumors are shown in Table 1.

**Table 1:** Demographic, surgical, and histopathologic features of patients undergoing resection of benign retroperitoneal nerve sheath tumors

	Age	Sex	Tumor Location	Tumor Size (cm)	Symptoms	Surgical Approach	Blood Loss (mL)	Surgery Duration (min)	Hospital Stay (days)	Histopathology	Residual Tumor
1	52	M	Lesser pelvis/presacral	16 × 14	Radicular pain (S1)	Intraperitoneal	2000	300	10	Neurofibroma, WHO Grade I	No
2	59	F	Lesser pelvis (left)	3 × 4	Radicular pain (L1)	intraperitoneal	300	180	13	Schwannoma, WHO Grade I	No
3	62	M	Paravertebral L5/retroperitoneal	5 × 5	Abdominal pain	retroperitoneal	500	200	12	Schwannoma, WHO Grade I	No
4	45	F	Presacral (left)	7.6 × 6	Asymptomatic	intraperitoneal	1000	300	7	Ganglioneuroma	Yes (2.1 × 1.8 cm)
5	61	M	Presacral	6 × 6	Asymptomatic	intraperitoneal	300	280	7	Schwannoma, WHO Grade I	No
6	48	F	L5 neuroforamen/prevertebral	9.4 × 4.1	L5 radicular pain	retroperitoneal	1000	310	14	Neurofibroma, WHO Grade I	No
7	25	F	Lesser pelvis (left)	8 × 7	Abdominal pain	intraperitoneal	800	320	5	Schwannoma, WHO Grade I	Yes (2.6 × 1.4 cm)
8	56	F	Presacral	5 × 4.5	L5 radicular pain	Retroperitoneal	700	400	6	Chronic hematoma (non-neoplastic)	No
9	55	F	Paraspinal L2/3 (right)	4 × 4.2	Local pain	Retroperitoneal	500	250	15	Schwannoma, WHO Grade I	No
10	35	F	Lesser pelvis	5 × 6	Asymptomatic	Retroperitoneal	350	220	7	Schwannoma, WHO Grade I	In S1 foramina
11	60	F	S1 neuroforamen/prevertebral/Presacral	7 × 6	S1 radicular pain	Retroperitoneal	800	270	14	Schwannoma, WHO Grade I	No
12	54		Lesser pelvis	4.5 × 5.1	Asymptomatic	Retroperitoneal	450	290	9	Schwannoma, WHO Grade I	No
13	46		Presacral	6 × 4	Local pain	Retroperitoneal	300	350	7	Neurofibroma, WHO Grade I	No

**Table 2:** Immunohistochemical results for schwannomas

ID	S100	SOX10	Ki67	PHH3 (Mitoses)	CD34	Vimentin
1	Positive	Positive	≤ 15%	Increased mitoses	Negative	Partially positive
2	Positive	Positive	1%	Not reported	Negative	Focal nerve fibers
3	Not reported	Positive	1%	Rare mitoses	Negative	Not reported
4	Positive	Positive	3%	Rare mitoses	Not reported	Negative
5*	Not reported	Not reported	Not reported	Not reported	Not reported	Not reported
6	Positive	Positive	1%	Rare mitoses	Negative	Negative
7	Positive	Positive	1%	Not reported	Negative	Not reported
8	Positive	Positive	1%	Rare mitoses	Negative	Negative
9	Positive	Positive	< 1%	Negative	Not reported	Positive
10	Positive	Positive	2%	Not reported	Negative	Not reported
11	Positive	Positive	3%	Not reported	Negative	Not reported

*In one patient who underwent surgery 12 years prior, immunohistochemical analysis was not performed

### Case example

**Fig. 4:**
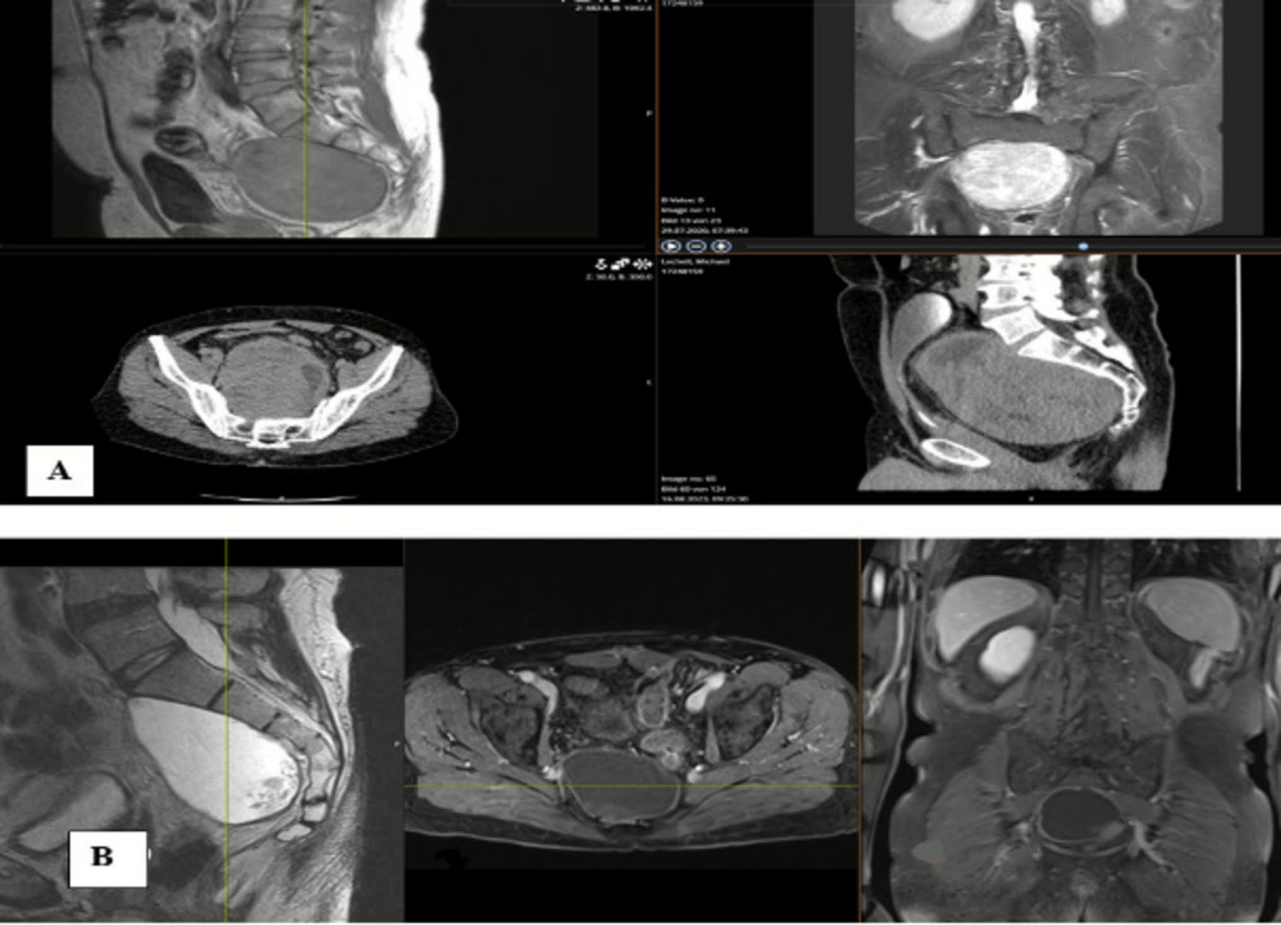
**A **CT and MRI showing Contrast enhanced large presacral lasion (16 × 14 × 12 cm) extending into the lesser pelvis. **B **Postoperative contrast-enhanced MRI demonstrated a fluid collection within the resection cavity, with no contrast uptake or residual tumor

A 52 year old male presented with right sided S1 radicular pain and hypesthesia. Contrast enhanced CT and MRI revealed a large presacral lasion (16 × 14 × 12 cm) extending into the lesser pelvis (Fig. 4A). CT-guided biopsy confirmed a WHO Grade I neurofibroma. Following multidisciplinary tumor board review, surgical resection was planned. Preoperative preparation included CT angiography, bilateral ureteral stenting, and routine laboratory studies. A midline lower abdominal laparotomy with transperitoneal approach was utilized due to the tumor’s size and retroperitoneal inaccessibility. The tumor capsule was dissected from adjacent vessels without injury. During surgery, following intracapsular debulking and tumor volume reduction, intraoperative neuromonitoring (IONM) identified the S1 nerve root traversing the tumor mass. Initial MEP thresholds of 15 mA were recorded, declining to 2 mA after meticulous dissection, confirming preserved nerve integrity. The procedure lasted 300 min with 2000 mL blood loss, managed via transfusion of 4 units packed red blood cells, fibrinogen, and 1 g tranexamic acid. Postoperatively, the patient reported complete resolution of radicular pain, with mild residual S1 hypesthesia and localized incision discomfort. Hemoglobin remained stable, and he was discharged on postoperative day 10. In three months follow-up MRI demonstrated a non-enhancing fluid collection at the resection site, with no evidence of residual or recurrent tumor (Fig. 4B).

## Discussion

In our cohort of 13 patients, surgical resection was found to be both safe and effective, even in cases involving large tumors. There were no postoperative complications observed in any patient. Although total tumor resection could not be achieved in three patients, the tumor mass was successfully reduced by approximately 80%. Given the typically slow growing and benign nature of these tumors, functional preservation (particularly of motor function) should be prioritized, even if a small residual portion of the tumor must be left in situ. This underlines the critical role of iONM in guiding surgical decisions. We emphasize the importance of detailed preoperative planning and comprehensive multidisciplinary discussion. Proper surgical preparation includes measures such as the placement of bilateral or ipsilateral double-J ureteral stents by the urology team to aid in the identification and mobilization of the ureters, thereby minimizing risk of iatrogenic injury during tumor dissection. Regarding the choice of surgical approach, we observed no significant difference in intraoperative course or immediate outcomes between retroperitoneal and intraperitoneal access. Vessel dissection from the tumor capsule was not technically challenging in our cases, but never the less, vascular surgeons should be informed and on standby to intervene, in case of accidental vascular damage.

### Tumor management

A significant correlation was observed between tumor size and intraoperative blood loss. This relationship is plausible, as larger tumors often demonstrate increased vascularity and require prolonged operative durations due to their anatomical complexity. Consequently, advanced preoperative preparations including securing blood products is critical for mitigating bleeding risks in such cases. Liu et al. compared laparoscopic and open approaches in the resection of schwannomas in 38 patients and found significant advantages associated with laparoscopy [13]. These included smaller incisions, reduced blood loss, faster recovery, and better postoperative neurological function. However, their study also reported postoperative complications: in the open group (8 cases, 40%) these included 4 urinary retention, 1 foot drop, 2 leg numbness, and 1 pelvic infection; in the laparoscopic group (3 cases, 16%), complications included urinary retention, leg numbness, and foot drop [13]. Lin et al. reported obturator nerve injury in two of six cases in their series (33%), resulting in activity limitation. We believe many of these complications are potentially avoidable through the use of intraoperative neuromonitoring and protective measures such as ureteral stenting [12]. Liu et al. also stated that due to the neurogenic origin of schwannomas, nerve fibers are often embedded in the tumor, making neurological sacrifice sometimes inevitable for complete resection [13]. However, in line with our findings and surgical philosophy, we argue that functional preservation should take precedence. In selected cases, retaining a minimal residual tumor may be preferable to compromising motor function. Fan et al. noted that while complete surgical resection with adequate margins remains the primary treatment for schwannoma, radical resection is not always necessary [6]. In the future, combining iONM with laparoscopic or robotic techniques may represent an ideal strategy for the resection of small, well-characterized lateral pelvic schwannomas. While some case reports and studies have documented successful total tumor resection without IONM and minimal postoperative complications [2–6, 8, 10–16], it is critical to contextualize these findings. Approximately 80% of schwannomas and neurofibromas arise from sensory nerves, where injury causes no motor deficits [12]. However, we emphasize that surgical resection should never be indiscriminate. In one case, a 45-year-old patient with no clinical symptoms was found to have a 9 × 7.5 cm lesion incidentally on imaging. The tumor arose from the sacral plexus and involved multiple motor nerve roots. Intraoperative neurophysiological monitoring revealed a 48% reduction in anal sphincter MEP amplitude and a 44% decrease in bilateral lower limb MEPs. To preserve functional nerve fibers while achieving maximal tumor debulking, we performed a subtotal resection, leaving a residual tumor measuring 5 × 4 cm. Although preoperative electrophysiological testing might have revealed abnormalities, the absence of symptoms makes this unlikely.

In a second case, our neuroradiology team and we initially suspected an L5 paravertebral schwannoma. However, both the intraoperative appearance and the definitive postoperative histology revealed an old, encapsulated hematoma. In hindsight, this operation could likely have been avoided by obtaining a preoperative CT-guided biopsy. Hughes et al. and Goh et al. emphasized in their study Imaging Features of Retroperitoneal and Pelvic Schwannomas that although rare, recognizing the characteristic imaging findings of schwannomas is critical for radiologists to avoid misdiagnosis as malignant tumors [8, 9]. Both studies agree that radiologic findings alone are usually nondiagnostic. We recommend including image-guided biopsy in the standard preoperative workup for comparable lesions. In situations where CT-guided biopsy is not feasible, intraoperative frozen section pathology plays a pivotal role. This is particularly crucial in suspected cases of malignancy such as sarcoma, where an en bloc resection may be warranted, even at the cost of sacrificing motor nerve fibers.

### Immunohistochemical markers

Our findings demonstrate that schwannomas exhibit consistent S100/SOX10 expression and low Ki67 proliferative indices (≤ 15% in cellular areas, otherwise < 1%), aligning with their benign WHO Grade I classification. Despite variable tumor sizes and locations, gross total resection with nerve preservation was achievable in 77% of cases using IONM, with no recurrences. Residual tumor showed no progression, underscoring the indolent biology of these tumors. In contrast, neurofibromas displayed patchier S100/SOX10 staining and slightly higher Ki67 (3%), though still within benign thresholds. Our results reinforce the utility of immunohistochemistry and multidisciplinary planning to avoid overtreatment of benign lesions. These outcomes mirror prior studies (Fan et al., Liu et al.) advocating nerve-sparing strategies over radical resection in slow-growing tumors [6, 13]. This evidence supports our surgical goals prioritizing function preservation, particularly in young patients.

## Conclusion

Surgical resection of retroperitoneal and pelvic schwannomas and neurofibromas, while technically challenging, is safe and effective when performed by experienced surgeons and multidisciplinary preoperative planning. None of our patients experienced postoperative complications, which may, in part, be attributable to the use of intraoperative neuromonitoring. However, comparative and prospective studies are recommended to further validate these findings.

## Data Availability

The datasets used and/or analyzed during the current study are available from the corresponding author on reasonable request.

## Abbreviations

*NF2*: Neurofibromatosis type 2
*NF1*: Neurofibromatosis type 1
*CT*: Computed tomography
*MRI*: Magnetic resonance imaging:
*CTA*: Computed tomography angiography
*MEPs*: Motor-evoked potentials
*EMG*: Electromyography
*IONM*: Intraoperative neuromonitoring
*ICU*: Intensive care unit
